# Using community analysis to explore bacterial indicators for disease suppression of tobacco bacterial wilt

**DOI:** 10.1038/srep36773

**Published:** 2016-11-18

**Authors:** Xiaojiao Liu, Shuting Zhang, Qipeng Jiang, Yani Bai, Guihua Shen, Shili Li, Wei Ding

**Affiliations:** 1 Laboratory of Natural Products Pesticides, College of Plant Protection, Southwest University, Chongqing, China

## Abstract

Although bacterial communities play important roles in the suppression of pathogenic diseases and crop production, little is known about the bacterial communities associated with bacterial wilt. Based on 16S rRNA gene sequencing, statistical analyses of microbial communities in disease-suppressive and disease-conducive soils from three districts during the vegetation period of tobacco showed that Proteobacteria was the dominant phylum, followed by Acidobacteria. Only samples from September were significantly correlated to disease factors. Fifteen indicators from taxa found in September (1 class, 2 orders, 3 families and 9 genera) were identified in the screen as being associated with disease suppression, and 10 of those were verified for potential disease suppression in March. *Kaistobacter* appeared to be the genus with the most potential for disease suppression. Elucidating microbially mediated natural disease suppression is fundamental to understanding microecosystem responses to sustainable farming and provides a possible approach for modeling disease-suppressive indicators. Here, using cluster analysis, MRPP testing, LEfSe and specific filters for a Venn diagram, we provide insight into identifying possible indicators of disease suppression of tobacco bacterial wilt.

As societal concerns regarding agricultural sustainability increase, soil is now considered a living system that is notably affected by microbial communities^1^. According to the classic definition, disease-suppressive soils are described as “soils in which the pathogen does not establish or persist, establishes but causes little or no damage, or establishes and causes disease for a while but thereafter the disease is less important, although the pathogen may persist in the soil^2^.” Conversely, severe disease broke out in the disease-conducive (nonsuppressive) soil^3^,^4^. Soils that are suppressive to diseases caused by soil-borne pathogens, such as *Rhizoctonia solani*^5^, *Fusarium oxysporum*^6^ and *Thielaviopsis basicola*^7^, have been described, and biological factors have been identified as the most important elements of this suppression^8^,^9^,^10^. It is well known that the interactions among soil microorganisms can induce the homeostasis of soil microbial communities and suppress soil-borne pathogens.

The rhizosphere is the narrow zone of soil that surrounds and is most strongly influenced by plant roots^11^. The importance of the rhizobacterial community to both plant growth and plant health has been studied for decades. Due to its high nutrient density, this region is the most dynamic location for microbial interactions, and it hosts beneficial microorganisms, soilborne pathogens, and competition among them^12^,^13^. The rhizobacterial community harbors tens of thousands of species that exert beneficial effects on plant growth and health, such as nitrogen-fixing bacteria, mycorrhizal fungi, plant growth promoting rhizobacteria (PGPR), biocontrol microorganisms, mycoparasitic fungi, and protozoa. Similarly, plant pathogenic microorganisms colonize the rhizosphere and cause plant diseases by breaking the protective microbial shield and overcoming the plant’s innate defense mechanisms^4^. The complexity and diversity of the organisms in the rhizosphere are essential for maintaining homeostasis in the ecosystem. Compared to the more variable efficacy of biocontrol agents in field inoculation experiments, the soil suppression of root disease has represented an ideal model by which plant protection is permanently implemented^14^.

Bacterial wilt of tobacco is one of the most economically important soilborne diseases in southern China. The causal bacterium, *Ralstonia solanacearum*, is a β-proteobacterium and is pathogenic to more than 200 plant species belonging to over 50 different botanical families^15^. This vascular pathogen is noted for its lethality, complex subspecies, wide host range, and broad geographic distribution^16^. Traditionally, resistant cultivars and chemical bactericides are the most frequently used practices for controlling tobacco bacterial wilt in China^17^. However, most disease-resistant tobacco cultivars decrease productivity, and undoubtedly, the use of chemical bactericides causes undesired side-effects on non-target organisms, including humans, and can pose risks to the environment. Therefore, the manipulation of natural microbial communities is considered one of the most promising strategies in increasing soil health for sustainable and integrated disease management^18^.

In this study, we sequenced the bacterial community from tobacco bacterial wilt disease-suppressive and disease-conducive soils to explore the possible indicators of disease suppression to tobacco bacterial wilt. All of the soils have a long history of tobacco monocropping. Previously, studies regarding the plant’s biocontrol mechanisms have focused on isolating beneficial microbial species and conducting growing experiments under greenhouse conditions^10^,^19^. However, the lack of consistency in controlling soilborne pathogens remains a major problem with microbial inoculants^20^. Furthermore, these microbial inoculants are always cultivable and represent a very small proportion of the microbial community of soil, whereas the difficult-to-culture taxa have been demonstrated to be dominant in natural environments^4^. In this report, we used the direct extraction method for 16S rRNA gene sequencing, which has enabled in-depth analyses of rhizobacterial communities from natural conditions^4^,^5^,^10^. Advances in next-generation sequencing have allowed us to characterize microbial communities at a broader range of spatial and temporal scales. Additionally, various algorithms used to assign taxa identity based on 16S rRNA gene sequences can support the identification of bioindicators for disease-suppressive soils.

## Results

### Comparison of soil microbial communities in March and September

**Figure 1:**
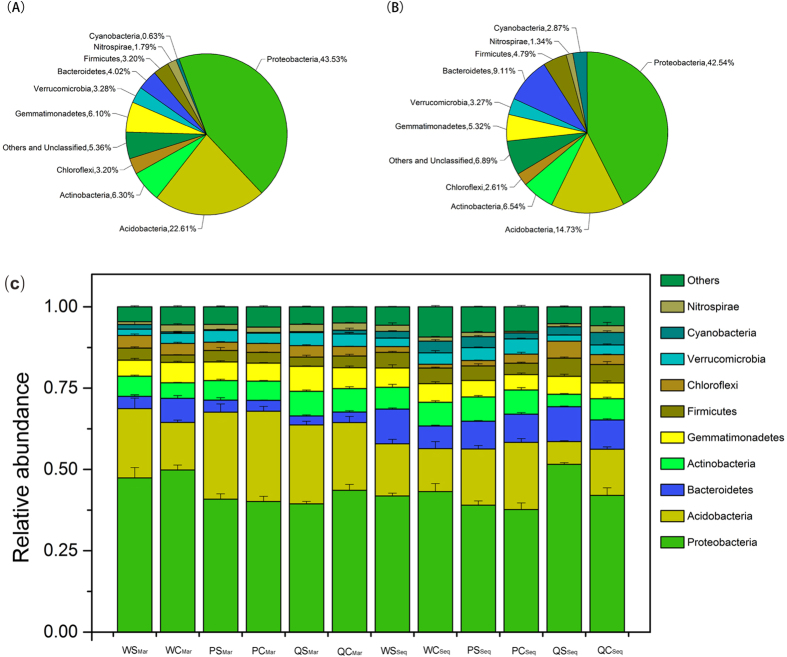
Overall distribution of microbes in March (A) and September (B) and bacterial diversity in the different conditions (C). Stacked bar graph representing the relative abundance (mean ± SE, n = 3) of major bacterial phyla in Wulong disease-suppressive (WS) and disease-conducive (WC) samples, Pengshui disease-suppressive (PS) and disease-conducive (PC) samples, and Qianjiang disease-suppressive (QS) and disease-conducive (QC) soil samples in March (subscripted Mar) and September (subscripted Sep).

Tobacco was grown from April to September 2014 in Chongqing, China. Across all tobacco vegetation in Chongqing, there was a massive outbreak of tobacco bacterial wilt in disease-conducive fields at harvest time, with over 50% of the tobacco being infected, but none of the plants in disease-suppressive fields were infected (Figure S1). A total of 13,809 bacterial and a few archaeal operational taxonomic units (OTUs) were detected in the soil microorganisms, 7989 in March (before the growing season) and 7738 in September (after harvest season) (Figure S2A). Although the count decreased by 251 OTUs, only 1918 OTUs remained the same (Figure S2A); that is, almost 75.21% changed during the cultivation period. However, based on the taxonomy to which the OTUs belong, nearly 67.53% of the March taxa remained unchanged in September (Figure S2B). Among the distribution of the predominant bacterial phyla, Proteobacteria was the dominant one, followed by Acidobacteria (Fig. 1A,B). Nevertheless, both of their relative abundances experienced a reduction from March to September, especially Acidobacteria, which decreased by almost 8%, whereas Bacteroidetes increased by 5.09%, Cyanobacteria by 2.24%, Firmicutes by 1.59%, Others and Unclassified by 1.53% and Actinobacteria by 0.24% (Fig. 1A,B).

The indices of richness and diversity were generally higher in September than in March (Table 1). In March, the number of OTUs per rarefied soil sample varied between 1497 (WS_Mar_) and 1953 (QS_Mar_). Sample QS_Mar_ showed the highest values for the Chao1 and Shannon indices (P < 0.05; Table 1). In September, there were no significant differences in the OTUs or the Chao1 index among samples.

**Figure 2:**
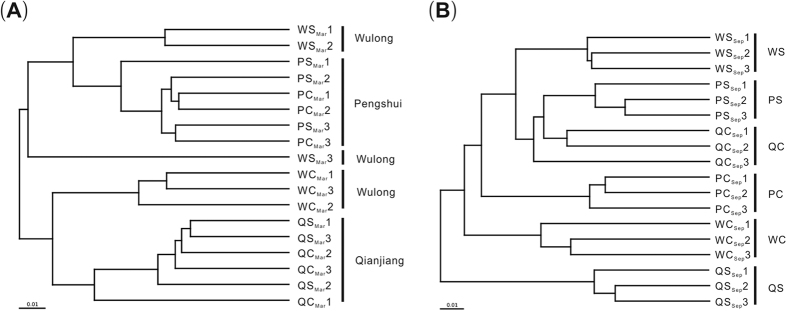
Cluster analysis based on weighted UniFrac distance in March (A) and September (B). Numbers 1 to 3 refer to the replicates of each treatment; other abbreviations as in Fig. 1.

Additionally, weighted (based on the abundance of taxa) UniFrac distance metrics were applied to estimate the β-diversity of samples^21^ (Fig. 2A,B), and a multi-response permutation procedure (MRPP) was used to test the significant difference of the β-diversity between the samples (Table S1). The clustering results showed that five clusters of samples mainly corresponded geographically to the three districts in March, except for the Wulong samples (Fig. 2A): two of the disease-suppressive samples from Wulong formed one cluster near to two other clusters, the samples from Pengshui and the other disease-suppressive sample from Wulong, and another two clusters were formed by the disease-conducive samples from Wulong and the samples from Qianjiang. The MRPP supported this geographic pattern with significant p values (p < 0.05) (Table S1). However, in September, due to the outbreak of tobacco bacterial wilt (Figure S1), six clusters of samples corresponded to the six soil groups based on the abundances of taxa (Fig. 2B). Moreover, the MRPP test revealed that the microbial diversity differed significantly based on both geographic and disease factors (p < 0.05) (Table S1).

### Comparison of soil microbial communities in disease-suppressive and disease-conducive soils

There was no clear tendency between disease-suppressive and disease-conducive samples in the soil physicochemical analyses (Table S2), except for the pH level: the pH values of disease-conducive soils were significantly higher than those of suppressive soils (Table S3). However, based on the Pearson correlation analysis, only Gemmatimonadetes (p < 0.01) negatively correlated to the pH of Qianjiang suppressive soils and Nitrospirae (p < 0.01) positively correlated to the pH of Wulong conducive soils (Table S3). Other bacterial communities were not significantly influenced by the pH value.

**Figure 3:**
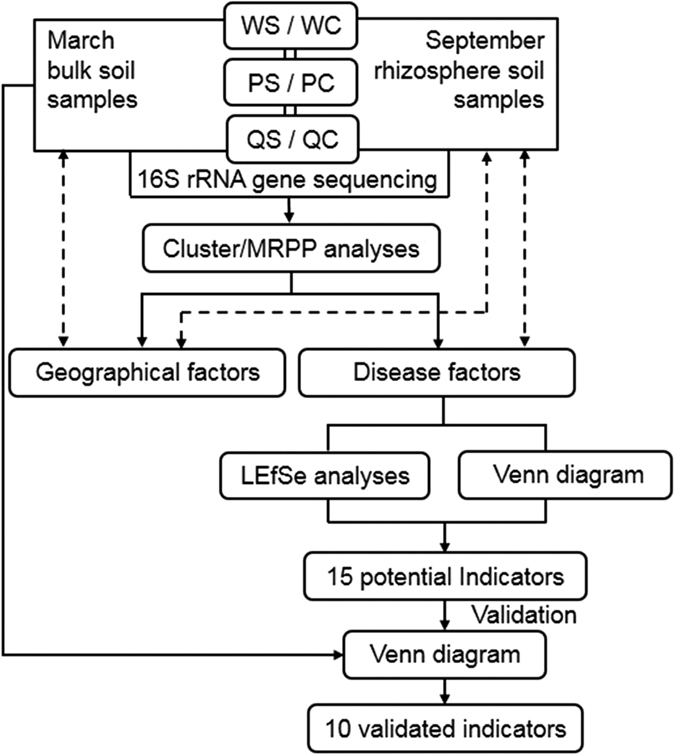
Flow chart of data analyses for the potential indicators among the March and September samples. Only sequencing data in September was significantly related to the disease factors and was consequently used to identify potential indicators through linear discriminant analysis (LDA) effect size (LEfSe) analyses and specific filters (S > C in all districts) by Venn diagramming. The selected 15 potential indicators were then verified by the sequencing data in March calculated under the same filters (S > C in all districts) by Venn diagramming. Dashed arrows represent significant correlations between samples and factors tested by MRPP (see Table S1); other abbreviations as in Fig. 1.

Comparing the OTUs in disease-suppressive soils with those in disease-conducive soils, there were 13,305 OTUs in the former and 12,992 OTUs in the latter, and the two groups shared 12,488 OTUs (Figure S2A). Similarly, although the distribution of the predominant bacterial phyla ranged from 0.23% for the Cyanobacteria in PS_Mar_ to 51.55% for the Proteobacteria in QS_Sep_ and from 0.23% for the Cyanobacteria in PC_Mar_ to 49.80% for the Proteobacteria in WC_Mar_ (Fig. 1C), no significant differences were found in the number of predominant bacterial phyla when comparing each district sample with the factor of disease involved. However, when considering the abundance of the detected taxa and the outbreak of disease, samples showed a significant correspondence to disease factor in September (Table S1). These results suggest that the microbial community in September is much more important for exploring the bacterial indicators associated with disease suppression. Therefore, the sequencing data from September were used as input data for two algorithms to screen for indicators, and if a bacterial taxon fit both criteria (detailed in the following section), it was chosen as a potential indicator (Fig. 3).

### Potential bacterial indicators of disease suppression toward tobacco bacterial wilt

**Figure 4:**
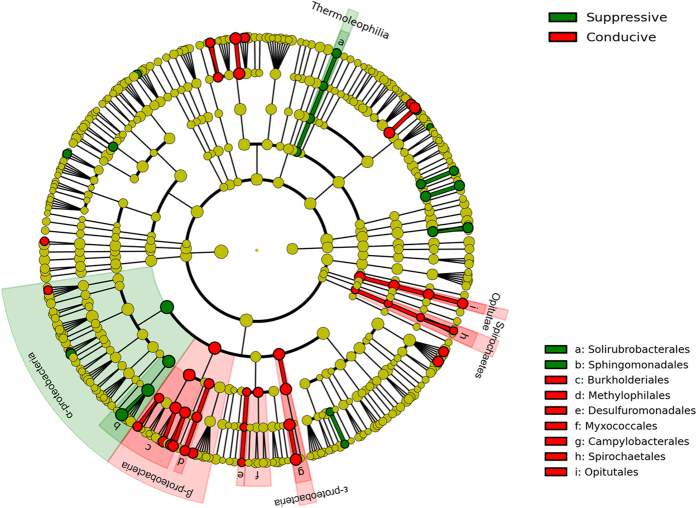
Discriminative taxa in suppressive and conducive groups in September. Significantly discriminant taxon nodes are colored and branch areas are shaded according to the highest-ranked variety for that taxon. For each taxon detected, the corresponding node in the taxonomic cladogram is colored according to the highest-ranked group for that taxon. If the taxon does not show significantly differential representation between sample groups, the corresponding node is colored yellow. Abbreviations were used for the significantly discriminant taxon at the order level. For the complete list of discriminant taxa and ranks used to generate this cladogram, see Dataset S1.

The samples in September were divided into two groups, disease-suppressive and disease-conducive soils, and the taxa were analyzed by linear discriminant analysis (LDA) effect size (LEfSe)^22^ to identify taxa as high-dimensional biomarkers with significant differential abundances between disease-suppressive and disease-conducive groups (Fig. 4 and Dataset S1). We identified 64 biomarkers, 22 suppressive taxa and 42 conducive taxa. Interestingly, no biomarkers were selected out at the phylum level; instead, 6 bacterial taxa at the class level were shown in Fig. 4. In the disease-suppressive groups, 2 lineages, α-Proteobacteria and Thermoleophilia, were prominent at both the class and genus levels. In comparison, members from 4 classes, β-Proteobacteria, ε-Proteobacteria, Opitutae and Spirochaetes, were identified in the disease-conducive soils (Fig. 4). Additionally, *Kaistobacter* was selected as the most prominent genus in disease-suppressive soils (Dataset S1). *Ralstonia,* the genus to which the pathogenic bacterium *Ralstonia solanacearum* belongs, was identified in the disease-conducive soils (logarithmic LDA score = 3.56) but was not the most common genus present (Dataset S1). However, the relative abundance of *Ralstonia* in the disease-conducive soils was twice that in the disease-suppressive soils (Figure S3).

**Figure 5:**
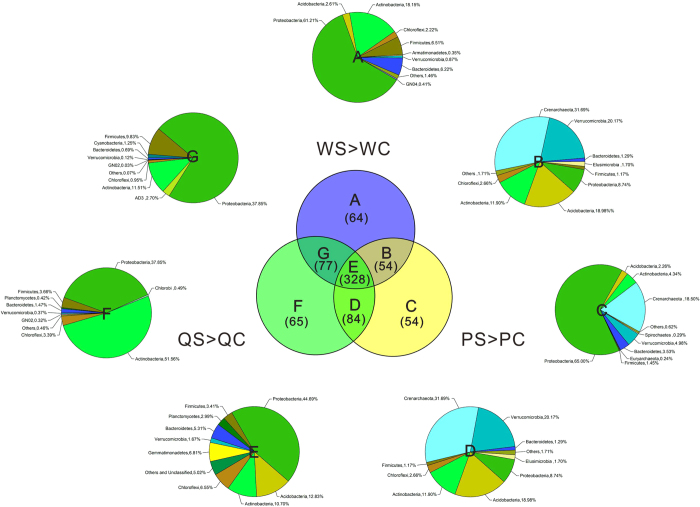
Bacterial and a few archaeal taxa associated with disease suppression. Shown are taxa that are more abundant in (i) Wulong suppressive soil (WS) than in Wulong conducive soil (WC), (ii) Pengshui suppressive soil (PS) than in Pengshui conducive soil (PC), and (iii) Qianjiang suppressive soil (QS) than in Qianjiang conducive soil (QC). Pairwise comparisons (n = 3) depict the compositions of the top 9 phyla. Numbers of taxa in each subset are in parentheses. The top 9 phyla that met all three criteria are shown in pie E and in dataset S2.

Additionally, inspired by Mendes^5^, we defined a rule to explore the most dynamic taxa associated with disease suppression in September: the detected OTUs were calculated on the basis of their taxonomy, and then the taxa were screened out and shown in intersections if they were more abundant in disease-suppressive soils than in disease-conducive soils for each district (Fig. 5 and Dataset S2). In theory, the detected OTUs should have been more abundant in suppressive soil than in conducive soil from each district (Fig. 5). Accordingly, 726 taxa were identified, among which Proteobacteria remained the most dominant phylum. Moreover, Actinobacteria was more abundant in Wulong and Qianjiang suppressive soils, and Crenarchaeota in Pengshui was not among the top 10 phyla in pie E (Fig. 5). Among the intersection of 328 taxa, almost half were from Proteobacteria (44.69%), followed by Acidobacteria (12.83%) and Actinobacteria (10.70%). *Kaistobacter* (6.04%) and Xanthomonadaceae (3.99%), both of which belong to the phylum Proteobacteria, were the most abundant genus and family, respectively (Dataset S2).

Comparing these observations from both algorithms to screen for our indicators, 15 taxa fit both criteria and were chosen as potential indicators (Table 2). These 15 indicators were 1 class, 2 orders, 3 families and 9 genera, 7 of which were from Actinobacteria, 5 from Proteobacteria, 2 from Acidobacteria and 1 from Firmicutes (Table 2). Among the 4 indicators with the highest scores, 3 came from Proteobacteria, particularly α-Proteobacteria, whose original abundances were relatively high in the Qianjiang disease-suppressive soils. Sphingomonadaceae was represented three times, among which the genus *Kaistobacter* had the highest original abundance (2.94% in WS, 2.93% in PS and 3.68% in QS) and abundance of specific filters in pie E (6.04%) and the second-highest logarithmic LDA score (=4.17) through LEfSe (Table 2). The class Alphaproteobacteria had the highest logarithmic LDA score (=4.50) as well as the second-highest original abundance (0.49% in WS, 0.52% in PS and 1.17% in QS) and abundance of specific filters in pie E (0.80%). Remarkably, at the genus level, *Catenulispora, Dermacoccus, Nocardia, Conexibacter* and *Actinocatenispora* were identified from *Actinobacteria*, although they together represented less than 0.45% of the bacterial taxa in the suppressive soils. Additionally, *Granulicella* from Acidobacteria and *Clostridium* from Firmicutes were identified as potential indicators at the genus level.

### Validation of the potential bacterial indicators in March

Based on cluster analyses with weighted UniFrac distance, the distribution of the bacterial community was found to be significantly related to geographic factors in March (Fig. 2, S3); however, these indicators should also indicate that the taxa were more abundant in disease-suppressive soils than in disease-conducive soils at each district in March (Table 2, Figure S4 and Dataset S3). It is therefore of paramount importance to verify these indicators through intersections with taxa under the defined rule (S > C in all districts) in March. Impressively, 10 indicators remained under this selection, and 2 Proteobacteria and 3 Actinobacteria were removed. Of the 9 selected genera, 5 were removed. However, *Kaistobacter* still had the highest abundance of specific filters in pie H (3.69%), while Acidobacteriaceae was the second most abundant (0.85%). Therefore, the indicators can be validated under the absence of disease outbreak; that is, 10 of the 15 indicators, especially *Kaistobacter*, are likely to indicate disease suppression toward tobacco bacterial wilt.

## Discussion

Disease-suppressive soils have been described worldwide, including *Rhizoctonia*-suppressive soils for sugar beets in the Netherlands^5^, *Fusarium* wilt-suppressive soils in Châteurenard and the Salinas Valley^6^, and *Thielaviopsis basicola*-mediated tobacco black root rot-suppressive soils in Morens^10^. The majority of studies on disease-suppressive soils have been restricted to individual specific beneficial microbes, ignoring the entire resident soil microbial communities^4^,^13^,^23^. Indeed, disease suppression is related to a global increase in soil microbial biomass because a large biomass has a greater opportunity to create a competitive environment that is deleterious for the pathogens^1^. Moreover, general suppression has been widely reported to suppress the growth or activity of soil-borne pathogens via nonspecific antagonism or biological buffering^2^,^24^,^25^. This is especially relevant in the case of soil amendment for the control of tobacco bacterial wilt, in which the antagonistic *Pseudomonas* showed no significant difference among treatments^17^. Accordingly, in the soil environment, microbial communities are diverse; we detected a total of 13,809 OTUs in this study alone. Those different types of microbial populations and their complex interactions may affect the specific beneficial microbes, plant growth, or even pathogens^10^,^26^.

To identify taxa that are indicative of soil suppressiveness, we compared tobacco rhizobacterial communities from six’ fields (disease-suppressive and disease-conducive to tobacco bacterial wilt in three districts) after the outbreak of tobacco bacterial wilt, notably where tobacco has been continuously cropped for more than 10 years. MRPP test based on weighted UniFrac distance showed that the microbial diversity was significantly related to the disease factor in September (Table S1). Additionally, the indices of richness and diversities in September were generally higher than those in March (Table 1), which guaranteed an abundance of taxa for selection. Therefore, we believe that the rhizosphere microbial community is most acceptable to be treated as a candidate indicator. We used a combination of LEfSe and specific filters (S > C in all districts) to identify the indicators. LEfSe was used to identify differentially abundant taxa between disease-suppressive and disease-conducive soils, which means that the indicators should be sufficiently discriminative in the samples. Although 22 high-dimensional biomarkers were identified for suppressive soils (Fig. 4 and Dataset S1), we defined another rule to further confirm their connection to disease suppression. The filters can enable a bias toward natural disease suppression for the detected taxa. Mendes *et al*.^5^ identified 17 OTUs as the most dynamic microbiota associated with disease suppression. However, to compare the microbiota identified by the two algorithms under the same level of taxa rather than OTUs, we therefore identified 328 taxa that fit the criteria (S > C in all districts), given that the LEfSe input was based on taxa. Eventually, if a bacterial taxon fit both criteria, it was then chosen as a potential indicator, and 15 taxa were identified as potential indicators.

The self-restoring capacity of soil microbiota during the period from the end of one production season to the beginning of the next may play an essential role in bacterial communities. It is therefore speculated that disease-conducive soils can become suppressive if agricultural measures, such as utilization of cover crops, deep plowing and biological pesticides, are conducted properly^6^,^27^. Although the samples obtained in March were bulk soils, they had been monocropped for over 10 years. To some extent, the samples in March represented the original status with or without pathogens but without disease. Therefore, the defined rule (S > C in all districts) would also apply to the samples in March. Ten indicators remained through the intersection of the two results. Hence, using the 15 bacterial indicators, especially the 10 that were validated in March, may be promising for practical management measures for manipulating the disease-conducive area before planting. This observation is supported by previous studies demonstrating that isolates from disease-suppressive soils can have substantial antibacterial activity^4^,^13^,^24^. However, less than 1% of the soil microbiota may be readily isolatable, whereas the remaining 99% of the microbes are viable but nonculturable (VBNC)^28^,^29^. Therefore, these indicators of disease suppression merit an assessment of their potential functional role in generating suppressive soils via culture-independent techniques.

Though a function cannot be directly attributed to bacteria identified with 16S rRNA gene sequencing, it is interesting to note that several indicators are known to include strains with biocontrol capacity^10^,^30^. Actinobacteria have been shown to represent a large fraction of microbial populations in root systems^31^ and are well-known as inhabitants of saprophytic soil^32^. They have the ability to produce a rich source of important natural products, especially antibiotics that suppress the growth and development of a wide range of soil-dwelling plant pathogens^1^,^33^,^34^,^35^. Previous studies have shown that 140 to 160 antibiotics have been used in human therapy and agriculture, 100 to 120 of which were produced by Actinobacteria^36^. Moreover, among the isolates that produced bioactive metabolites, the rare Actinomycetales represented 26%, among which *Nocardia* was one of the highest producers^37^. Many *Nocardia* species have shown potent capacities to metabolize aliphatic and aromatic toxic hydrocarbons, natural or synthetic polymers, and other widespread environmental pollutants that are not readily degradable^34^. In addition to *Nocardia, Catenulispora acidiphila* was demonstrated to produce the class III lantipeptide catenulipeptin, a 27-amino acid peptide that contains two labionin bridges and has no antimicrobial activity but was able to stimulate aerial mycelium formation in surfactin-treated *Streptomyces coelicolor*^38^. Presumably, *Catenulispora* have the potential to be indicators along with certain beneficial microorganisms.

The most abundant genus associated with disease suppression in our study, *Kaistobacter,* has been described as showing significantly greater abundance in replanted soil than in new soil^39^. Moreover, *Kaistobacter* has been reported to have the ability to biodegrade both EPTC (S-ethyldipropylthiocarbamate) and atrazine in soils^40^. There is no information available about its possible functional role in disease suppression^41^, but the family to which *Kaistobacter* belongs, Sphingomonadaceae, has been reported to be more prevalent in the tobacco rhizosphere in suppressive soil^10^. Certain strains from the family Sphingomonadaceae are closely associated with nitrogen fixation^42^. This may be relevant for plant health in terms of resistance to pathogen attacks. The class α-Proteobacteria was also a discriminant taxon as an indicator for disease suppression in the current study. It has been reported that α-Proteobacteria played a prominent role in soil suppression of *Rhizoctonia solani*^5^. Moreover, the majority of the sequences suggested that α-Proteobacteria was the most abundant class in the rhizosphere soil^43^,^44^. α-Proteobacteria is one of the most abundant classes of Proteobacteria, which is known to play important roles in carbon, nitrogen, and sulfur cycling^45^. Additionally, Acidobacteria, another indicator identified in our study, has also been shown to occur in diverse environments as a dominant bacterial group^46^. Using acidobacterial subgroups as bioindicators has been suggested to expand the possibilities for managing the effects of agricultural soil in the greater Amazon area^9^. However, members of this phylum have been difficult to isolate *in vitro*, and many basic features of their biology and functional roles in the soil have not yet been determined.

Although pure culture analysis of soil microorganisms has revealed that they are capable of controlling several diseases *in vitro*^13^,^47^, studies based on 16S rRNA gene sequencing have extensively redefined and expanded our knowledge of soil microbial diversity and have begun to reveal a much greater part of the uncultured fraction of the soil microflora^5^,^10^. However, we cannot yet link the entire microbiome to the cause of disease suppression in disease suppressive soils, and thus identifying several important microbes that have a great potential to indicate the disease suppression seems more practicable. With the help of varied community analysis approaches, an initial framework of selecting disease-suppressive indicators was formed. Revealing these 15 indicators functionally responsible to disease suppression at the molecular level is a major challenge. Therefore, two important steps would be currently to expand this research that: (i) verify those 15 indicators in soils with different treatments in pot experiments (e.g., inoculating pathogens in different concentrations with different plants especially from *Solanaceae*); and (ii) discover the mechanism by which they participate in disease suppression through metagenomics. Only if we understand how these indicators function in the disease suppressive processes, we will be able to use such a model to pick out the potential indicators directly from numerous sequencing data, which may be used to the indicator exploration of most soil-borne disease in the future.

## Materials and Methods

### Site and sampling

Soil samples were collected in March (before the tobacco growing season) and September (after the harvest season) 2014, from Wulong (29°23.177′N, 107°24.518′E), Pengshui (29°10.008′N, 107°57.913′E), and Qianjiang (29°15.765′N, 108°42.777′E) in Chongqing, China. A map of these three distinct locations was drawn using ArcGIS 9.3 (http://www.esri.com/software/arcgis/) and is shown in Figure S5. These locations are the primary sites of tobacco production in Chongqing. The distance between disease-suppressive and disease-conducive soil samples in each field is less than 10 kilometers (Figure S5). Fertilizers and pesticides were applied in the three districts 3 times from April to June under the standards established by Chongqing Tobacco Corporation. No herbicides were used during the growing season.

As there was no tobacco planting in March, we gathered bulk soil samples from 15 random sites across each field at a depth of 5–25 cm, where the interaction between roots and the rhizosphere microbial communities occurred intensively after planting. For the September rhizosphere samples, the complete root systems of three plants at the same fields in March were combined as a composite sample after removing loosely adherent soil by vigorous shaking. Soils were sieved (2-mm mesh) to remove plant debris, pooled in sterile plastic bags, homogenized thorough hand mixing in triplicate and immediately transferred to the laboratory and stored at ambient temperatures (approximately 2–4 °C) within 24 hours of sampling. Physical and chemical analyses on each soil sample from March were performed using standard methods in the soil analysis laboratory of Southwest University, China (Tables S2 and S3).

### Disease incidence in the fields

The area of each location is 0.2 ha, containing approximately 3300 tobacco plants. The disease incidence of tobacco bacterial wilt was calculated in each replicate at the harvest time. Because the disease-suppressive fields were not infected, we only described the conducive ones (Figure S1).

### DNA extraction and construction of sequencing libraries

Microbial DNA was isolated from 0.4 g of soil per extraction using standard protocol for Omega Biotek Soil DNA Kit (Omega Biotek, USA). DNA concentration and purity was monitored on 1% agarose gels. DNA was diluted to 1 ng/μl using sterile water. Duplicates were performed for each sample, and the resulting DNA extracts from each sample were mixed for PCR experiments. PCR amplifications were conducted with primers 515 forward (5′-GTGCCAGCMGCCGCGGTAA-3′) and 806 reverse (5′-GGACTACHVGGGTWTCTAAT-3′), which amplify the V4 region of the 16S rDNA gene^48^.

PCR amplification consisted of an initial denaturation at 98 °C for 1 min, 30 cycles of denaturation at 98 °C for 10 s, annealing at 50 °C for 30 s, and elongation at 72 °C for 60 s, and a final extension at 72 °C for 5 min. PCR amplifications were performed in triplicate using 30 μL reactions with 15 μL of Phusion High-Fidelity PCR Master Mix (New England Biolabs), 0.2 μmol of forward and reverse primers, and 10 ng of template. Amplified products were run on 2% agarose gels for identification; samples with bright main bands between 200 and 250 bp were chosen for further experiments. Amplicons were combined at roughly equal amplification intensity ratios, purified using the GeneJET Gel Extraction Kit (Thermo Scientific) and submitted to the next-generation sequencing laboratory of Novogene Bioinformatics Institute, Beijing, China, for Illumina paired-end library preparation, cluster generation, and 250-bp paired-end sequencing. Because the samples were collected at different times, the sequencing was conducted in March and September, respectively. The raw reads have been deposited into the NCBI short-reads archive database under accession number SRP066888.

### Bioinformatics and statistical analysis of sequencing data

Raw Illumina fastq files were demultiplexed, quality filtered, and analyzed using QIIME v1.6.0 49 software (Quantitative Insights Into Microbial Ecology). Reads were truncated at the first site of the length (default setting = 5) from continuous low-quality score (default setting ≤ 10).

Sequence prefiltering (discarding sequences with <70% pairwise identity to any reference sequence) and reference-based OTU picking were performed using a representative subset of the Greengenes bacterial 16S rRNA gene database^50^ and filtered to remove incomplete and unannotated taxonomies^51^. Sequences with ≥97% similarity were assigned to the same OTUs. We picked representative sequences for each OTU and used a QIIME-based wrapper of the Ribosomal Database Project (RDP) classifier^52^ to annotate taxonomic information for each representative sequence.

Comparison of overall microbial distribution in March and September was conducted on the relative abundances of phyla that were calculated using OTUs on the basis of taxonomy using the Origin 9.0 software to generate the pie chart and histogram (Fig. 1). Categorized into the groups March suppressive/conducive and September suppressive/conducive, OTUs and the taxa that were calculated using OTUs on the basis of taxonomy are presented separately using the website^53^ http://bioinfogp.cnb.csic.es/tools/venny/index.html to generate the Venn diagram (Figure S2). The relative abundance of *Ralstonia,* detected only in the September samples, is presented separately in Figure S3 using Origin 9.0 software.

Perl scripts were used to analyze α- (within-sample species richness) and β- (between-sample community dissimilarity) diversity. We calculated the Chao1^54^ and Shannon indices as α-diversity. One-way analysis of variance (ANOVA) was performed to distinguish the differences among bacterial community compositions, and Pearson correlation analysis was used to test correlations between the pH value and the top 12 bacterial communities, using SPSS Statistics 17.0 (SPSS, Chicago, Illinois, USA). Unweighted Pair Group Method with Arithmetic Mean (UPGMA) Clustering was calculated by QIIME for weighted UniFrac distance^21^,^55^ to represent β-diversity. UPGMA Clustering is a hierarchical clustering method using average linkage and can be used to interpret the distance matrix. For deeper data mining of microbial diversity for the differences between the samples, a multi-response permutation procedure (MRPP) was carried out using R software version 3.3.1 with the package vegan for the significance test.

Based on the significance test, only rhizosphere soil (samples in September) communities were significantly related to disease factors. Therefore, we used the sequencing data in September to identify potential indicators, and the sequencing data in March was used to verify the selected indicators. We used two algorithms to complete this selection; if a bacterial taxon met both criteria, it was then chosen as a potential indicator. One of the algorithms is linear discriminant analysis (LDA) effect size (LEfSe)^22^, which ensured the selection of taxa with significantly different abundances between disease-suppressive and disease-conducive soils. LEfSe employs the factorial Kruskal–Wallis sum-rank test (α = 0.05) to identify taxa with significantly different abundances between categories (using one-against-all comparisons), followed by LDA to estimate the effect size of each feature with differential abundance (logarithmic LDA score = 2.0). Significant taxa were used to generate taxonomic cladograms illustrating differences between sample classes on the website http://huttenhower.sph.harvard.edu/galaxy. The taxonomic levels were limited from domain to genus in case of distraction from redundant data. The other algorithm is the analysis of specific filters (S > C in all districts) by Venn diagram, which enabled a bias toward natural disease suppression for the detected taxa. OTUs from September were calculated on the basis of taxonomy. Only taxa that were more abundant in disease-suppressive soils than in disease-conducive soils at each district were included. The selected taxa were then evaluated for membership in each section through the website^53^ http://bioinfogp.cnb.csic.es/tools/venny/index.html.

The same rule (S > C in all districts) used in the Venn diagram in September was applied to OTUs from March, and the taxa in the center intersection were then used to verify the indicators (Figure S4). Validated indicators were those selected indicators that could also be shown in the center intersection of the March Venn diagram.

## Additional Information

**How to cite this article**: Liu, X. *et al*. Using community analysis to explore bacterial indicators for disease suppression of tobacco bacterial wilt. *Sci. Rep.* **6**, 36773; doi: 10.1038/srep36773 (2016).

**Publisher’s note:** Springer Nature remains neutral with regard to jurisdictional claims in published maps and institutional affiliations.

## Supplementary Material

Supplementary Information

Supplementary Dataset S1

Supplementary Dataset S2

Supplementary Dataset S3

## Figures and Tables

**Table 1:** Richness and diversity (mean ± SE, n = 3) of bacterial rRNA gene fragment sequences in soil samples.

	OTUs (97%)	Chao1 index	Shannon index
Mar.			
WS_Mar_	1497.00 ± 104.48a	1998.94 ± 402.18a	9.49 ± 0.06ab
WC_Mar_	1898.33 ± 42.33bc	3427.74 ± 129.94b	9.85 ± 0.06b
PS_Mar_	1690.67 ± 45.24abc	2962.93 ± 94.19ab	9.09 ± 0.20a
PC_Mar_	1615.67 ± 94.38ab	2364.18 ± 301.54ab	9.54 ± 0.07ab
QS_Mar_	1953.00 ± 5.57c	3314.53 ± 30.85b	9.91 ± 0.01b
QC_Mar_	1896.67 ± 44.28bc	3076.01 ± 259.68ab	9.80 ± 0.04b
Sep.			
WS_Sep_	3213.67 ± 64.83a	4296.00 ± 231.22a	9.81 ± 0.02bc
WC_Sep_	3264.33 ± 87.86a	4011.15 ± 217.11a	10.04 ± 0.01d
PS_Sep_	3269.33 ± 85.99a	4211.28 ± 294.09a	10.01 ± 0.05cd
PC_Sep_	2886.67 ± 150.65a	3641.28 ± 363.15a	9.66 ± 0.08b
QS_Sep_	3016.67 ± 54.87a	4250.37 ± 209.21a	9.39 ± 0.04a
QC_Sep_	3282.33 ± 38.96a	4552.77 ± 144.64a	9.86 ± 0.05bcd

Values followed by different letters indicate statistically significant differences (P < 0.05, Student-Newman-Keuls).

**Table 2:** Indicators of rhizobacterial communities for disease suppression (mean ± SE, n = 3).

Taxon of Indicators					Relative Abundance (%)			LDA score (log10)	Abundance (%) in Pie E	Abundance (%) in Pie H
Phylum	Class	Order	Family	Genus	WS_Sep_	PS_Sep_	QS_Sep_			
Proteobacteria	Alphaproteobacteria	Sphingomonadales	Sphingomonadaceae	*Kaistobacter*	2.94 ± 0.04	2.93 ± 0.12	3.68 ± 0.21	4.17	6.04	3.69
Proteobacteria	Alphaproteobacteria				0.49 ± 0.09	0.52 ± 0.19	1.17 ± 0.06	4.50	0.80	0.21
Actinobacteria	Thermoleophilia	Solirubrobacterales			0.67 ± 0.04	0.42 ± 0.01	0.49 ± 0.03	2.75	0.69	0.50
Proteobacteria	Alphaproteobacteria	Sphingomonadales	Sphingomonadaceae		0.41 ± 0.01	0.48 ± 0.05	0.62 ± 0.02	4.06	0.37	0.18
Acidobacteria	Acidobacteria	Acidobacteriales	Acidobacteriaceae		0.46 ± 0.05	0.31 ± 0.05	0.33 ± 0.03	3.20	0.46	0.85
Acidobacteria	Acidobacteria	Acidobacteriales	Acidobacteriaceae	*Granulicella*	0.16 ± 0.02	0.18 ± 0.00	0.27 ± 0.02	3.20	0.36	0.18
Actinobacteria	Actinobacteria	Actinomycetales	Catenulisporaceae	*Catenulispora*	0.26 ± 0.07	0.13 ± 0.02	0.15 ± 0.02	3.19	0.28	0.04
Proteobacteria	Alphaproteobacteria	Sphingomonadales			0.10 ± 0.03	0.12 ± 0.00	0.11 ± 0.03	4.06	0.07	None
Actinobacteria	Actinobacteria	Actinomycetales	Dermacoccaceae	*Dermacoccus*	0.02 ± 0.00	0.01 ± 0.00	0.10 ± 0.01	2.86	0.06	None
Actinobacteria	Actinobacteria	Actinomycetales	Nocardiaceae	*Nocardia*	0.04 ± 0.01	0.03 ± 0.01	0.05 ± 0.01	2.80	0.06	0.01
Actinobacteria	Thermoleophilia	Solirubrobacterales	Conexibacteraceae		0.05 ± 0.00	0.02 ± 0.00	0.00 ± 0.09	2.75	0.03	0.11
Actinobacteria	Thermoleophilia	Solirubrobacterales	Conexibacteraceae	*Conexibacter*	0.04 ± 0.01	0.02 ± 0.00	0.01 ± 0.00	2.75	0.04	None
Firmicutes	Clostridia	Clostridiales	Clostridiaceae	*Clostridium*	0.02 ± 0.00	0.01 ± 0.00	0.01 ± 0.00	2.81	0.01	0.05
Proteobacteria	Gammaproteobacteria	Alteromonadales	Shewanellaceae	*Shewanella*	0.00 ± 0.00	0.00 ± 0.00	0.01 ± 0.00	3.21	0.01	None
Actinobacteria	Actinobacteria	Actinomycetales	Micromonosporaceae	*Actinocatenispora*	0.00 ± 0.00	0.00 ± 0.00	0.00 ± 0.01	3.08	0.00	None
