# Supplementary material for: Using community analysis to explore bacterial indicators for disease suppression of tobacco bacterial wilt
Source: Sci Rep. 2016 Nov 18;6:36773. doi: 10.1038/srep36773 (PMC5114674; doi:10.1038/srep36773)
Supplement: Supplementary Information [file srep36773-s1.pdf]

Using community analysis to explore bacterial indicators for disease suppression of tobacco bacterial wilt

Xiaojiao Liu, Shuting Zhang, Qipeng Jiang, Yani Bai, Guihua Shen, Shili Li, and Wei Ding*

Laboratory of Natural Products Pesticides, College of Plant Protection, Southwest University, Chongqing, China

Supplementary Information

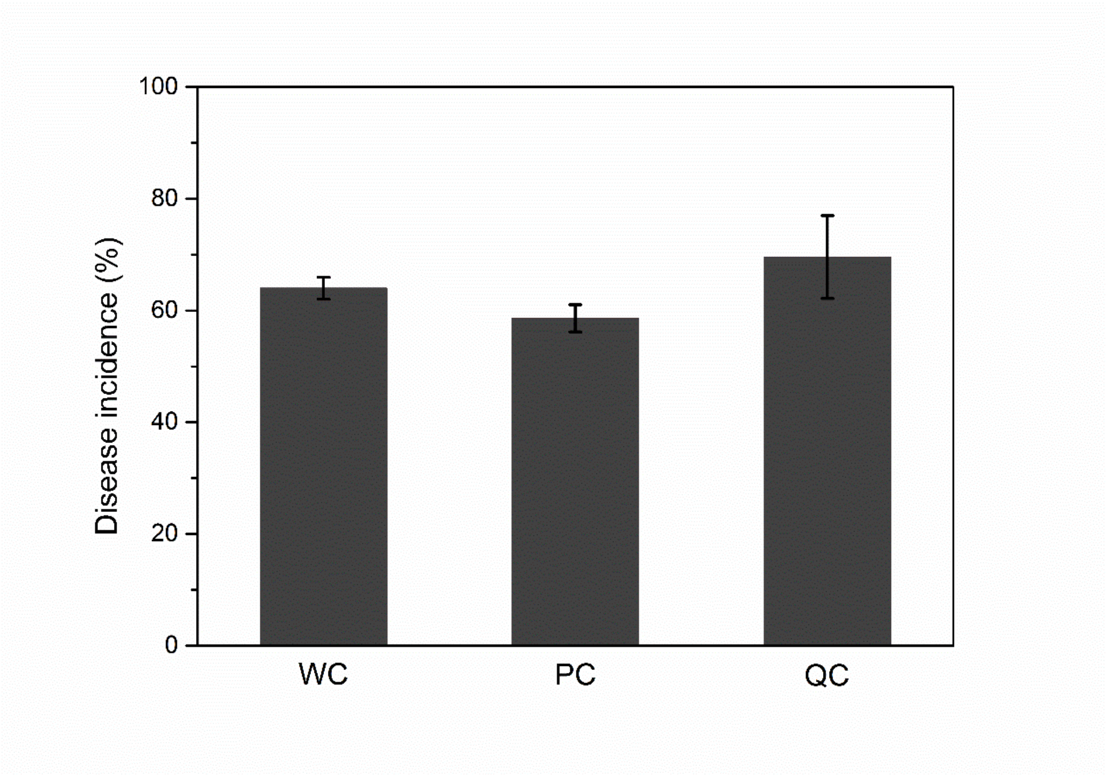

Figure S1 Disease incidence at three sites conducive to tobacco bacterial wilt at harvest time. WC, Wulong conducive sites; PC, Pengshui conducive sites; QC, Qianjiang conducive sites.

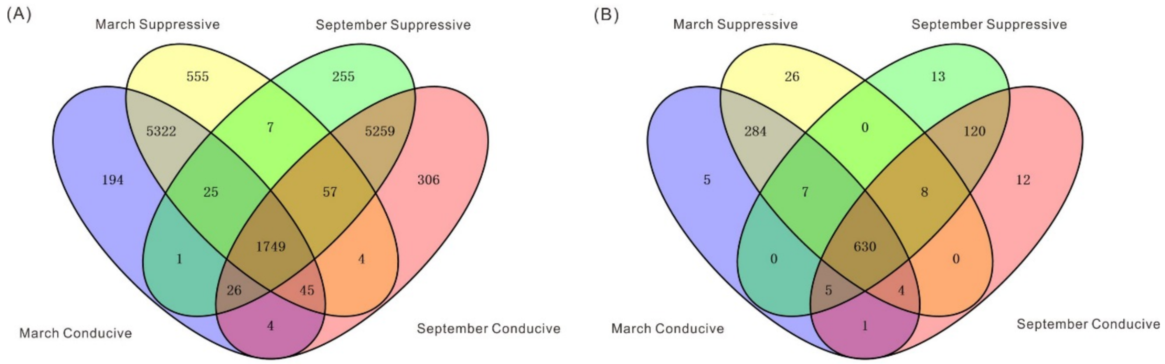

Figure S2 Comparisons of disease-suppressive and disease-conducive soils in March and September based on the OTUs (A) and taxa (B).

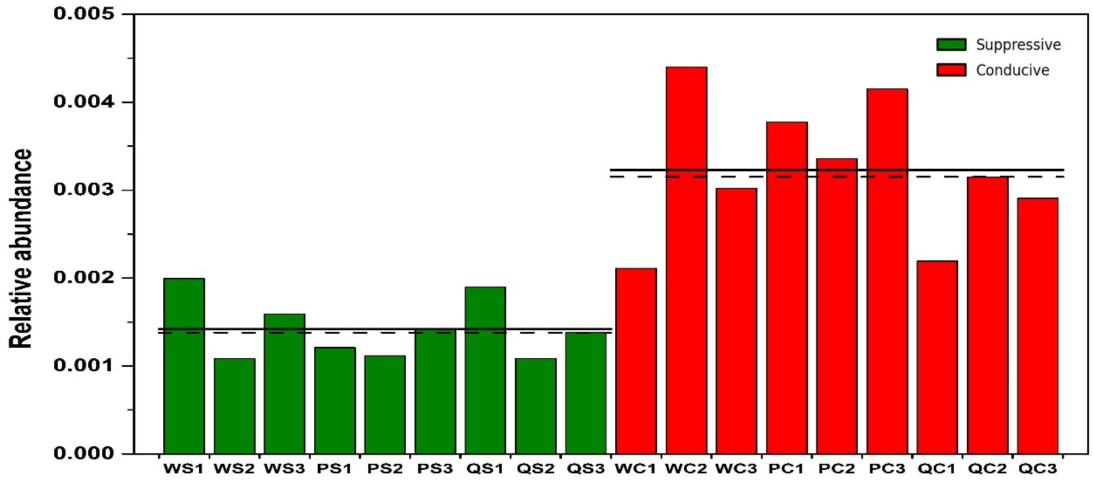

Figure S3 The relative abundance of *Ralstonia* in September. Solid horizontal lines represent the subclass means, while dotted horizontal lines represent the subclass medians. Numbers 1 to 3 refer to the replicates of each treatment; other abbreviations are as in Figure 1.

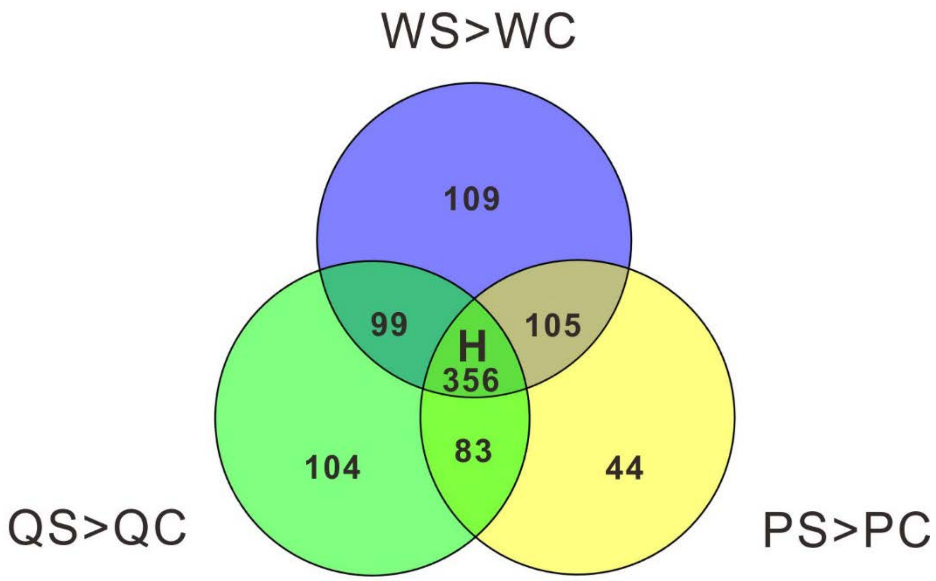

Figure S4 Bacterial and a few archaeal taxa associated with disease suppression in March. Numbers shown are taxa that are more abundant in (i) Wulong suppressive soil (WS) than in Wulong conducive soil (WC), (ii) Pengshui suppressive soil (PS) than in Pengshui conducive soil (PC), and (iii) Qianjiang suppressive soil (QS) than in Qianjiang conducive soil (QC). Pairwise comparisons (n = 3) depict the compositions of the top 9 phyla. The top 9 phyla that met all three criteria are shown in pie H and in dataset S3.

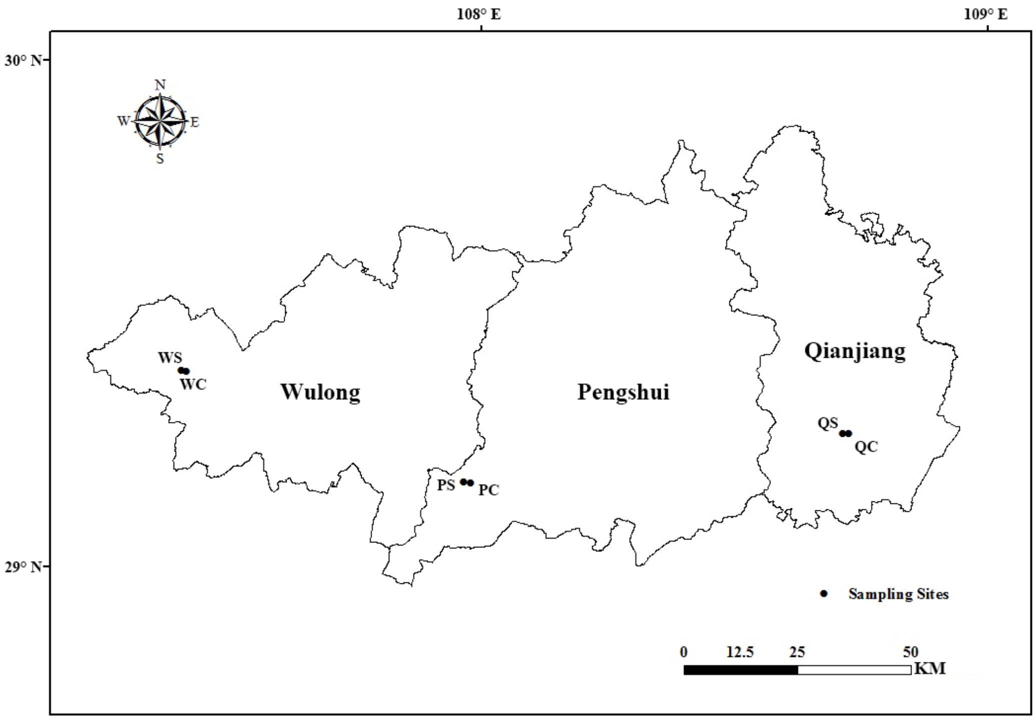

Figure S5 Sampling map in Wulong, Pengshui and Qianjiang. ArcGIS 9.3 (http://www.esri.com/software/arcgis/) was used to generate this map. Abbreviations as in Figure 1.

Table S1 The significance test of the geographic factor and disease factor on the overall microbial community structure in March and September.

Data sets	March		September	
	δ	*P*	δ	*P*
Geographic factor				
Wulong-Pengshui	0.1682	0.002	0.0808	0.008
Wulong-Qianjiang	0.1864	0.002	0.0748	0.026
Pengshui-Qianjiang	0.2521	0.002	0.1836	0.002
Disease factor				
Suppressive-Conducive	0.0097	0.251	0.0486	0.017

The MRPP test is a non-parametric multivariate analysis based on dissimilarities among samples. The statistic δ is the overall weighted mean of within-group means of the pairwise dissimilarities among sampling units. The significance test evaluates the fraction of permuted δ that is less than the observed δ. The *P* value is derived from the corresponding significance test.

Table S2 Soil Physicochemical Data.

	WS	WC	PS	PC	QS	QC
Organic matter (g/kg)	28.9	32.9	24.6	26.1	20.2	17.3
Total C (g/kg)	16.8	19.1	14.3	15.1	11.7	10.1
Total N (g/kg)	1.6	1.75	2.05	1.43	1.4	1.16
Total P (g/kg)	1.1	1.17	1	0.733	0.888	1.02
Total K (g/kg)	11.2	11	11.4	13.4	13.1	10.3
Clay < 0.002 (mm, %)	41	40	43	37	38	34
Silt 0.002-0.02 (mm, %)	24	24	24	28	27	20
Sand 0.02-2 (mm, %)	35	36	33	35	35	46

Abbreviations as in Figure 1.

Table S3 Pearson correlation analyses of pH (mean ± SE, n = 3) and bacterial community.

	WS	WC	PS	PC	QS	QC
pH	4.63±0.88a	6.67±0.15d	5.57±0.03b	6.13±0.07c	4.60±0.15a	5.77±0.88b
Bacterial community	Pearson correlation coefficient					
Proteobacteria	-0.834	-0.867	0.254	0.953	-0.613	0.035
Acidobacteria	0.965	0.988	-0.01	-0.917	-0.512	0.177
Actinobacteria	-0.992	0.885	-0.523	0.822	-0.912	-0.988
Gemmatimonadetes	0.82	0.995	-0.92	-0.862	-1.000**	0.1
Bacteroidetes	-0.943	-0.679	0.291	0.647	0.901	0.641
Verrucomicrobia	-0.904	-0.545	-0.418	-0.941	0.993	0.565
Firmicutes	-0.957	0.901	0.341	0.41	0.965	-0.901
Chloroflexi	0.354	0.993	-0.302	0.041	0.801	-0.845
Nitrospirae	0.416	1.000**	0.298	-0.739	0.4	0.182
Planctomycetes	0.299	0.733	-0.969	0.227	0.493	-0.171
AD3	-0.065	0.622	-0.734	-0.597	-0.657	0.581
Cyanobacteria	-0.861	-0.871	-0.195	-0.997	-0.032	0.093

Values followed by different letters indicate statistically significant differences ($P < 0.05$, Student-Newman-Keuls).

**Denotes a p value of < 0.01, which is typically regarded as significant.
